# New Perspectives on Canned Fish Quality and Safety on the Road to Sustainability

**DOI:** 10.3390/foods14010099

**Published:** 2025-01-02

**Authors:** Antónia Juliana Pais-Costa, António Marques, Helena Oliveira, Amparo Gonçalves, Carolina Camacho, Helga Coelho Augusto, Maria Leonor Nunes

**Affiliations:** 1 Interdisciplinary Centre of Marine and Environmental Research (CIIMAR/CIIMAR-LA), University of Porto, Terminal de Cruzeiros do Porto de Leixões, 4450-208 Matosinhos, Portugalamparo@ipma.pt (A.G.); ccamacho@ciimar.up.pt (C.C.);; 2 Division of Aquaculture, Upgrading and Bioprospection, Portuguese Institute for the Sea and Atmosphere, I.P. (IPMA, I.P.), 1495-165 Algés, Portugal; 3 Cofisa, S.A., Terrapleno do Porto de Pesca—Gala, 3090-735 Figueira da Foz, Portugal; helgacaugusto@cofisa.pt

**Keywords:** canned seafood, histamine, allergens, marine biotoxins, bisphenols, toxic elements, PAHs, POPs, new canned products

## Abstract

Canning extends the shelf life of seafood products while preserving their quality. It is increasingly considered a more sustainable food processing method due to the primary fishing methods used for key species and the lower energy costs compared to the production of fresh and frozen fish. However, canning can change key components, allow some contaminants to persist, and generate undesirable compounds. This review revisits the effects of canning on product quality and highlights the potential hazards that may compromise safety. It also examines emerging trends in product development, particularly novel formulations aimed at optimizing nutritional value while maintaining safety standards without compromising sustainability. Overall, the quality of most canned seafood meets industry requirements, for example, with improvements in processing strategies and strict safety protocols, leading to reduced histamine levels. However, data on marine biotoxins and microplastics in canned seafood remain limited, calling for more research and monitoring. Environmental contaminants, along with those generated during processing, are generally found to be within acceptable limits. Product recalls related to these contaminants in Europe are scarce, but continuous monitoring and regulatory enforcement remain essential. While new formulations of canned fish show promise, they require thorough evaluation to ensure both nutritional value and safety.

## 1. Introduction

Canning is a traditional processing method that plays a crucial role in preserving seafood [1]. Continuous improvements of the canning process have significantly enhanced the environmental sustainability of the fishing industry. By extending the shelf life of seafood and minimizing spoilage, canning helps reduce food waste [2]. Additionally, most canned fish products are sourced from fishing practices that minimize bycatch and environmental impacts. Some companies provide consumers with information on where and how the fish was caught, enabling informed decisions that support responsible fishing practices [3].

According to the Food and Agriculture Organization (FAO), canned seafood accounted for 11% (approximately 19.5 million tons) of global fisheries’ production in 2020. Canned seafood is produced from various raw materials, primarily tuna, salmon, sardines, and mackerel, and includes headed and gutted small fish; filets or portions; chunks, flakes, or shreds; and, to a lesser extent, shellfish. Recently, a variety of culinary preparations, such as salads or complete meals, have been introduced to the market [4]. The canned seafood industry is expected to grow due to increasing demand for convenient seafood, supported by enhanced global distribution infrastructures that allow for extended storage and consumption without compromising strict food safety standards. The global market for canned seafood is projected to grow at an annual rate of 6.2% from 2024 to 2031, with the tuna sector, which held a 40% market share in 2022, expected to remain dominant during this period [5].

The production of canned fish involves several steps, including pre-processing, filling, sealing, sterilization, cooling, and storage. These steps vary depending on the raw materials and desired end-product characteristics. Standard sterilization typically involves maintaining high constant retort temperatures (120–130 °C) for extended periods (usually over 60 min) to ensure quality and minimize processing time. This process adheres to required F0 values (the thermal lethality time needed to eliminate all microorganisms by exposing them to a temperature of 121.1 °C) to ensure product safety and commercial stability [1]. While the canning process is well established, recent suggestions advocate for improvements, such as using variable retort temperature profiles instead of constant high temperatures, to reduce processing time and energy consumption while optimizing product quality and safety [1]. These improvements could also contribute to sustainability.

Safety concerns with canned fish remain, including the presence of histamine [6,7], bisphenol A (BPA), and BPA analogs like bisphenol S (BPS) (e.g., [8,9,10]). Other potential hazards include toxic elements (e.g., [11]), marine biotoxins [12], microplastics [13], and thermostable allergens such as parvalbumin [14].

The development of novel canned seafood products is on the rise, including those with an innovative filling medium and/or enriched with bioactive compounds (e.g., [4]).

This article provides a comprehensive analysis of recent research aimed at understanding the impact of the canning process on product quality, identifying common safety issues, and assessing the development of new, safe canned seafood options. It addresses key concerns, such as toxic elements, microplastics, bisphenols, and marine toxins, where data are limited. Additionally, it examines trends in innovative filling media and bioactive ingredient-enriched canned products, and how the industry has adapted to evolving policies. This review fills a crucial gap in the literature on canned seafood safety, offering a critical assessment and identifying areas for further research.

## 2. Materials and Methods

The bibliographic search was conducted across the PubMed, ScienceDirect, Web of Science, Google Scholar, and Scopus databases, for the period between 2010 and 2023.

**Figure 1:**
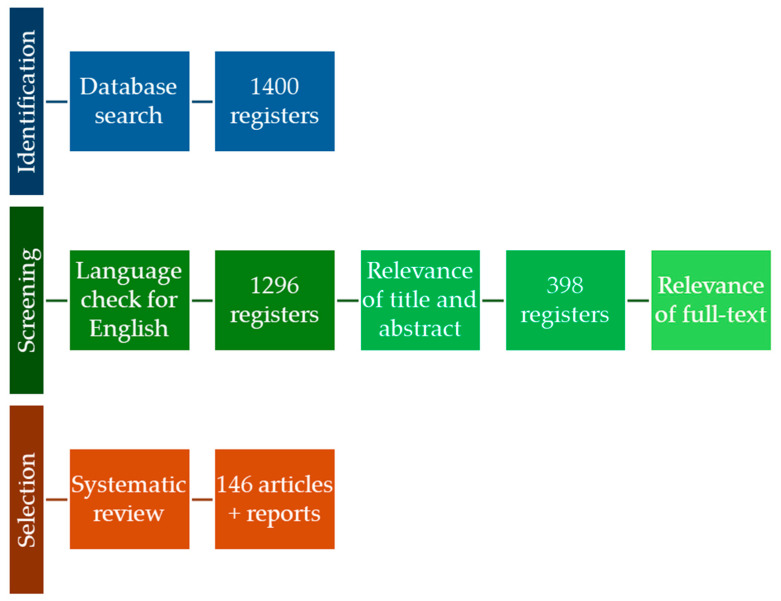
The flowchart of the selection method of references for inclusion in the literature review.

The search terms were adapted for each database to optimize the retrieval of relevant studies. Regarding study classification, articles were initially screened based on the relevance of their titles and abstracts. Those passing this preliminary screening underwent a full-text review. The classification of studies was based on their alignment with the review’s objectives, focusing on the relevance and depth of information they provided. The selection criteria were that the (i) full text was available, and (ii) full text was published in English. Theses, letters to editors, and papers presented at conferences were excluded. The flowchart in Figure 1 summarizes the review methodology.

The following search terms were used: “canned”, “canning”, “fish”, and “seafood”, in combination with other terms such as “safety”, “quality”, “hazards”, “histamine”, “biotoxins”, “bisphenol A”, “BPA”, “BPA migration”, “BPA exposure”, “bisphenol A diglycidyl ether”, “BADGE migration”, “BADGE derivatives migration”, “BADGE exposure”, “BADGE derivatives exposure”, “BPA-analogs”, “bisphenol S”, “BPS”, “BPA-analogs migration”, “BPA-analogs exposure”, “toxic metals”, “cadmium”, “Cd”, “lead”, “Pb”, “mercury”, “Hg”, “tin”, “Sn”, “polycyclic aromatic hydrocarbons”, “PAHs”, “halogenated persistent organic pollutants”, “POPs”, “dioxins”, “dioxin-like PCBs”, “PCBs”, “perfluorinated alkyl substances”, “PFASs”, “aluminum”, “microplastics”, “thermostable allergens”, “new formulations”, “new products”, and “new ingredients”.

## 3. Results and Discussion

### 3.1. Impacts of Seafood Canning Chain on Quality

The canning process has significantly improved over the years with the adoption of Hazard Analysis and Critical Control Points (HACCP) principles [15] and the installation of turnkey lines specifically designed for efficient seafood processing. However, certain issues, such as the quality of raw materials and the canning process itself, remain critical throughout the value chain.

After harvesting, seafood raw materials deteriorate quickly due to microbial activity and various degradation pathways caused by their chemical composition, nearly neutral pH, and high water content [16]. As a result, preservation processes are necessary to slow down this deterioration [17,18]. Most species intended for canning require freezing and frozen storage as essential preservation strategies, as they are caught in large quantities, need to be available year-round, are frequently caught in distant fishing areas, and often need to be stored for long periods before processing [17,19,20].

The canning process, especially the time and temperatures used during sterilization, continues to be a major research focus for strategies that minimize time and energy consumption while maximizing quality and safety. Although most facilities use a constant retort temperature, the dynamic optimization of variable retort temperature profiles has been suggested as a more effective approach to reduce energy costs without compromising quality and safety [1,21,22]. However, the canning process and long-term storage of the final product under commercial conditions can induce changes (e.g., browning, oxidation, nutrient loss) that may affect taste and shelf life [23,24]. The type of filling medium (e.g., brine, olive oil, sunflower oil) also influences sensory attributes, chemical composition, and the quality evolution of canned seafood during storage [25,26,27,28,29,30,31]. Gómez-Limia et al. [32] found that the fatty acid profile of European eels changes during the canning process, often resembling that of the filling medium. Domiszewski [28] studied how canning temperature affects eicosapentaenoic (EPA) and docosahexaenoic (DHA) levels in canned herring, mackerel, and sprats. The study reported that sterilization at 115 °C caused up to a 7.5% loss of fatty acids in oil, while about 10% of fatty acids transferred to the tomato sauce. Dantas et al. [33] observed that the filling medium influences the fatty acid profile, with increased levels of fatty acids from the oil (e.g., oleic and linoleic acids) and decreased levels of polyunsaturated fatty acids like EPA and DHA. The authors also found that filling mediums, particularly brine, play a significant role in forming cholesterol oxidation products, likely due to pro-oxidizing elements such as salt and enhanced heat transfer in brine. Additionally, Gómez-Limia et al. [32] noted that canned eel in sunflower oil retained higher antioxidant capacity and vitamin E content after one year of storage compared to those in olive oil and olive oil with spices.

Regarding amino acids, the filling medium and storage significantly affect the contents depending on the specific amino acid. Gómez-Limia et al. [32] reported decreases in methionine and glycine and increases in proline after the sterilization of canned swordfish in various filling mediums (olive oil, spiced olive oil), relative to the raw eels. However, sterilization did not cause changes in the essential amino acid index (IEAA). Based on the calculated IEAAs, the quality of the amino acid proteins in the final product (canned samples stored for 12 months) decreased in the following order: canned eels in sunflower oil > olive oil > spiced olive oil. The study further revealed that changes in the amino acid content of canned seafood depend on the filling medium and storage time.

The current findings suggest no specific trend for water, protein, and lipid content, as well as for labile and other compounds (vitamins, lipids, minerals, fatty acids, amino acids, cholesterol) in canned seafood products. However, the nutritional value does not seem to be significantly affected by these variations [24].

### 3.2. Hazards in Canned Seafood

There is a growing concern that the safety of marine species is under pressure due to the increase and spread of biological contaminants caused by global climate change, as well as the accumulation of microplastics and chemical contaminants from anthropogenic activities. These hazards can accumulate in marine resources, potentially exceeding the tolerance limits for human consumption. Bridging the knowledge gaps between the spread of dangerous agents in the marine environment and their effects on seafood will be necessary [34].

Canned seafood products can present several biological, physical, and chemical hazards if the raw materials are not properly monitored and/or are mishandled, processed, or stored incorrectly. The main potential hazards associated with canned seafood consumption include histamine, thermostable allergens, marine biotoxins, bisphenols, toxic elements, and microplastics.

#### 3.2.1. Biological Hazards

##### Histamine

Food safety criteria and regulations regarding histamine, the most problematic biogenic amine in canned seafood products, vary significantly among countries worldwide [7]. Fish species associated with high potential levels of histamine typically belong to families such as Scombridae, Clupeidae, Engraulidae, Coryphaenidae, Pomatomidae, and Scombresosidae [35]. In 2019, Mercogliano and Santonicola [36] reviewed factors influencing histamine production in the tuna supply chain, identifying storage temperature as the most critical control measure.

Regarding histamine levels, many countries have adopted the regulations set by the European Commission (EC) [35,36] or the FAO/World Health Organization (FAO/WHO) [37] (see Table 1). When histamine limits are exceeded, regulatory bodies implement mandatory product recalls.

Recent studies on canned tuna, mackerel, and sardine from various markets revealed a wide average range of histamine concentrations, fluctuating from 4.6 ± 2.8 to 98.10 ± 5.18 mg/kg [55,56,57,58,59,60,61,62,63,64,65,66,67,68,69], which are below the accepted limits proposed by the EC and FAO/WHO for adverse health effects. However, histamine concentrations varied widely, with maximum concentrations sometimes exceeding the legal limit (ranging from below the limit of detection (LOD) to 216.9 mg/kg). Currently, canned fish products from species prone to histamine accumulation exhibit low histamine levels. This is a result of continuous improvements in canning processes, driven by widespread adoption of HACCP principles and national and international regulations to ensure canned seafood safety.

Overall, studies suggested that oil, brine, and tomato sauce fillings may increase the risk of histamine poisoning [60,66]. However, no significant variance in histamine levels was observed across different filling mediums (e.g., oil or natural) [59,68].

##### Thermostable Allergens

Canned fish is sometimes recommended due to its benefits, as some individuals with fish allergies can tolerate it, though the exact mechanisms for this tolerance remain unknown [14]. A possible explanation includes a decrease in allergenicity caused by sterilization, which may result in conformational changes in allergenic proteins.

Parvalbumin, a thermally stable and calcium-binding protein, is the most common fish allergen [70]. Some studies show that parvalbumin content in canned tuna varies depending on the process used (e.g., [14]). An average decrease of 25% in parvalbumin concentration was observed after the canning process of fish from the Southern Hemisphere [71]. However, the immunoglobulin E (IgE) reactivity of parvalbumin increased after thermal treatment. Data on the presence of parvalbumin in 29 commercially canned tuna products from 13 different brands, in various filling mediums (sunflower oil, olive oil, spiced, and light tuna), indicated that its presence was influenced by the filling medium, thermal conductivity, calcium content, and acidity of ingredients. According to Blickem et al. [72], undeclared tuna allergens were classified as one of the primary reasons for commercial tuna recalls in the United States between 2002 and 2020. Additionally, a study on canned fish products (salmon, tuna, and sardines prepared in salt water) revealed that all products contained fish allergens (related to parvalbumin, tropomyosin, and/or collagen), suggesting that canned fish products can trigger allergic reactions in fish-allergic patients due to significant IgE binding to these proteins [73]. Consequently, an allergy advisory regarding seafood ingredients should be included on canned fish labels.

Based on these findings, canned fish products may not be safe for all fish-allergic individuals. It is recommended that the immunogenicity of canned fish be further investigated [74].

##### Marine Biotoxins

Marine biotoxins are produced by certain species of microalgae as a defense mechanism or by-product of their metabolism. Shellfish, including mussels and clams, are filter feeders that accumulate toxin-producing microalgae, making them the main route of exposure to regulated marine toxins for consumers [75]. Fish and microalgae species from tropical and subtropical regions can also accumulate emerging toxins like tetrodotoxins and ciguatoxins [76], potentially posing a future risk to consumers of canned seafood products. The European Union (EU) regulations permit the use of bivalve mollusks if the initial level of contamination with Paralytic Shellfish Poisoning toxins exceeds the limit of 80 µg/100 g but is below 300 µg/100 g [77,78]. However, shellfish must undergo rigorous processing operations sequentially.

There is limited information regarding the levels of other marine toxins in canned seafood. Blanco et al. [79] reported reduced Diarrheic Shellfish Poisoning toxin levels in non-commercial contaminated mussels following canning by 24.1% for okadaic acid (OA) and 42.5% for Dinophysistoxin (DTX)-2, though the toxicity remained nearly unchanged. In contrast, Rodríguez et al. [80] found that DTX-3 in seafood is eliminated during canning (121 °C) and that different heat treatments (e.g., mild steaming at 100 °C for 5 min, industrial steaming at 105 °C for a minimum of 2 min) had varying effects on Diarrhetic Shellfish Toxin analogs, with some remaining stable and others decreasing. Garcia et al. [81] assessed the effect of canning non-commercial contaminated bivalves and gastropods on lipophilic toxins. They reported that the canning process reduces toxin content by up to 15% and facilitates the interconversion of Pectenotoxin (PTX)-group toxins into PTX-2sa in bivalves. Additionally, they found no redistribution of toxic analogs of OA-, PTX-, and yessotoxin-group toxins between visceral and non-visceral tissues, nor any detection of esterified analogs (acyl-OA/DTX-1) in bivalves and gastropods after canning.

These findings indicate that the health risks of marine biotoxins vary depending on heat treatment and toxin analogs.

#### 3.2.2. Chemical Hazards

##### Bisphenols

Epoxy resins used to coat the inside of cans are produced from the condensation of epichlorohydrin and BPA, forming bisphenol A diglycidyl ether (BADGE) and its derivatives [82]. These compounds, including BPA and BADGE, can migrate from the coating into food, posing potential health risks [83]. BPA is an endocrine-disrupting compound linked to various health issues (e.g., [84,85,86]), and research on BADGE and its derivatives suggests similar health concerns [83,87]. Another compound of concern is Cyclo-di-BADGE (CdB), a by-product of epoxy resin production that comprises BPA and BADGE [44].

To protect human health, organizations have established specific migration limits (SML) into food for certain bisphenols (Table 1). In 2023, the European Food and Safety Authority (EFSA) published an opinion lowering the Tolerable Daily Intake (TDI) of these substances from 4 to 0.002 µg/kg bw/day [10]. In response, the EC drafted an initiative to ban the use of BPA in food contact materials by the first quarter of 2024 [9,10]. The diploma remains to be approved.

In response to stricter BPA regulations, manufacturers are turning to BPA analogs like bisphenol S (BPS). However, there is limited information regarding their safety compared to BPA. Research suggests that these analogs may lead to adverse health effects that can be similar to or exceed those associated with BPA [87,88].

Canned food, particularly seafood, is a significant pathway for human exposure to BPA [40,89]. The most recent review on canned seafood was conducted in 2016, and since then, the SML value for BPA has been further restricted (Table 1). Recent studies assessing BPA, BADGE, and their analogs in canned seafood from various origins and with diverse filling mediums reveal that BPA contamination levels in European products generally comply with updated SML standards (Table 1 and Table 2). However, elevated BPA levels were detected in three out of nine samples of canned tuna from Turkey, ranging from 0.05 to 0.10 mg/kg of food [90], and in one out of two samples of tuna from Spain, with levels reaching 0.41 mg/kg of food [91]. Conversely, canned seafood from non-European countries exhibited much higher BPA concentrations than the permitted European SML (Table 2). Regarding BADGE and its derivatives, concentrations generally remained below the respective European SMLs (Table 1 and Table 2). Samples where CdB levels were accessed (canned tuna) showed values exceeding the German acceptable limit (Table 2), suggesting the need for further research on the potential health risks of this chemical. Canned tuna was the most analyzed product, with BPA concentrations ranging from below LOD to 0.41 mg/kg of food. Interestingly, this range is similar to that reported in the review of Repossi et al. [92], despite stricter SML standards. Overall, the solid fraction (SF) exhibited higher BPA values than the liquid fraction (LF) [93,94]. This trend was also reported by Repossi et al. [92].

BPS concentrations in canned products generally remained below the SML, with the exception of the study by Gálvez-Ontiveros et al. [91], which identified BPS in Europe at a concentration of 0.19 mg/kg of food, significantly exceeding the accepted SML.

Data on estimated daily BPA intake indicate that levels fall below the European TDI of 4 μg/kg bw/day but exceed the new TDI of 0.002 μg/kg bw/day, raising serious concerns for consumers (Table 3). Intake levels of BPS and BADGE, along with its hydroxyl derivatives, are below the current safety limits. However, for CdB, studies report higher estimated daily intake values than those suggested by Biedermann et al. [46]. The estimated daily intake values found in Europe for BPA underscore the urgent need for manufacturers to enforce stricter controls over packaging materials and transition toward safer packaging systems to ensure consumer safety.

##### Toxic Elements

Despite stringent control and safety regulations, the potential for canned fish to be contaminated with toxic elements remains a concern. These contaminants can be present throughout the fish’s lifecycle, from handling and transportation to processing and canning stages [98]. Due to their toxicity, most studies on toxic elements primarily focus on cadmium (Cd), lead (Pb), and mercury (Hg). Aluminum (Al) and tin (Sn) were also considered in this review due to the potential of migration from the can material to the food, which could compromise the safety or quality of the canned product [99,100].

To safeguard human health, regulatory agencies worldwide have established limits for toxic element contamination in seafood (Table 1). Overall, concentrations of Al, Cd, Hg, and Sn in canned fish across various studies were below the Maximum Permissible Limits (MPLs) (Table 4). However, there were notable exceptions. For Al, Kosker et al. [101] found concentrations up to 14.45 mg/kg (mean: 6.77 mg/kg food) in 15 out of 29 samples of canned tuna purchased in Europe, considerably exceeding the EU SML of 5 mg/kg food. In non-European countries, de Lima et al. [102] found Al concentrations significantly higher than the EU SML in 8 out of 16 samples of canned tuna acquired in Brazil (Table 4). Ababneh and Al-Momani [103] found mean concentrations of Cd in canned tuna from Jordan up to 2.5 times higher than the MPLs (0.54 ± 0.05 and 0.63 ± 0.04 mg/kg). Similarly, Massadeh et al. [104] documented mean Cd concentrations exceeding MPLs in canned sardines and tuna from Jordan (0.42 ± 0.07 and 0.47 ± 0.03 mg/kg, respectively). For Pb, most samples either fell below or slightly exceeded the MPLs. Nonetheless, some studies found Pb concentrations significantly surpassing the MPLs. In Europe, Mol [100] reported Pb concentrations as high as 3.05, 2.88, and 3.05 mg/kg in canned tuna, sardines, and mackerel, respectively, far exceeding the MPLs of 0.3 mg/kg. However, mean Pb concentrations in these products were generally within the EU MPLs (0.209 ± 0.580 mg/kg for canned tuna, 0.284 ± 0.605 mg/kg for canned sardines, and 0.313 ± 0.877 mg/kg for canned mackerel). In Asia, Sadighara et al. [105] detected mean PB concentrations of 0.71 mg/kg in one sample of canned tuna, while Massadeh et al. [104] reported mean Pb concentrations of 2.8 and 2.5 mg/kg in canned tuna and sardines, respectively. Sobhanardakani [106] also reported mean Pb levels of 0.75 ± 0.65 mg/kg in canned fish (tuna and common kilka). Finally, no correlation was observed between the concentration of toxic elements and either the filling medium or the fish species, based on the data gathered for this review.

The existing data on the estimated daily intake of toxic elements from canned seafood, sourced from both EU and non-EU countries, indicate that these levels fall below the respective TDI (Table 1). As a result, they do not appear to pose a concern for consumers [93,101,105,106,107,108].

**Table 4:** Concentration range (mg/kg) of cadmium (Cd), mercury (Hg), lead (Pb), aluminum (Al), and tin (Sn) in canned tuna from various origins. Limit of detection (LOD). Total number of samples analyzed (*n*).

Origin	Species	*n*	Toxic Metals					References
			Cd	Hg	Pb	Al	Sn	
European countries	Tuna	*279*	<LOD^*^–110	<0.001–0.29	<0.007–3.05	<LOD*–14.45	<0.001–0.19	[100,101,108,109,110]
	Sardines	*100*	<0.001–113	<0.001–0.45	-	-	<0.001–0.16	
	Mackerels	*53*	<0.001–0.12	<0.001–0.21	<0.001–3.05	-	<0.001–0.39	
non-European countries	Tuna	*457*	<LOD^*^–0.63	0.01–0.79	0.02–2.80	<LOD^*^–47.33	4.9–157.90	[93,102,103,104,105,106,107,111,112,113]
	Sardines	*201*	<LOD^*^–0.42	-	<LOD^*^–2.50	<LOD^*^–5.12	-	
	Fish	*200*	0.02–0.15	0.02–0.18	0.04–1.60	-	-	

^*^ LOD: Cd ≤ 0.0004 and Al ≤ 0.004 mg/kg for European countries; Cd ≤ 0.0006, Pb ≤ 0.0051, and Al ≤ 0.001 mg/kg for non-European countries.

##### Other Contaminants

Emerging contaminants, such as polycyclic aromatic hydrocarbons (PAHs) and halogenated persistent organic pollutants (POPs), can pose risks to human health [114]. However, there is limited literature on their presence in canned fish products. To safeguard human health, regulatory agencies have established limits for some of these contaminants (Table 1).

The presence of PAHs in canned fish generally arises during the pre-canning processing of raw materials, such as smoking, drying, or grilling. However, the resulting products are mostly intended for niche markets [115]. Some studies reported the levels of PAHs in canned smoked fish, including sprat, mackerel, herring, and shellfish [110,116,117,118,119,120]. As expected, smoked canned fish samples generally showed higher levels of PAHs compared to unsmoked samples [110,118]. Some studies also indicate that maximum levels of benzo[a]pyrene (BaP) and PAH4  (sum of benzo[a]antracene or BaA, BaP, benzo[b]fluoranthene, or BbFA and chrysene or CHR) exceed the permissible limits of 0.005 mg/kg and 0.03 mg/kg, respectively, established by the EU [44]. For example, Drabova et al. [118] found that levels of BaP and PAH4 in canned smoked sprats, collected from the Czech market, were above the permissible limit (with means of 0.009 and 0.05 mg/kg, respectively). Zachara et al. [117] found that canned smoked sprats available on the Polish market had PAH4 levels of up to 0.073 mg/kg, but the mean concentration was 0.010 mg/kg, which falls within the permissible limit set by the EU Regulation.

POPs include three major groups of chemicals: chlorinated, fluorinated, and brominated. These chemicals enter the food chain through the die, accumulate in fish, and are eventually transferred to consumers through fish consumption [121]. Regarding chlorinated chemicals, Afolabi et al. [122] investigated the levels of polychlorinated biphenyls (PCBs), dioxins, and dioxin-like PCBs in canned mackerel, tuna, and sardines from Nigerian markets. The authors found the following levels: Σ [dioxins] at 0.002, 0.004, and 0.003 mg/kg; Σ [dioxins and dioxin-like PCBS] at 0.003, 0.005, and 0.004 mg/kg; and Σ [PCBs] at 0.0008, 0.0006, and 0.001 mg/kg. Only the Σ [PCB] concentrations were below the EU limits (Table 1). Vali Mohammadi et al. [123], Drabova et al. [118], and El Morsy et al. [119]) reported PCB levels in canned seafood from markets in Iran, Czech Republic, and Egypt, respectively, all below the EU limit (0.075–0.300 mg/kg). As for fluorinated chemicals, particularly perfluorinated alkyl substances, only the study by Hrádková et al. [124] was found. The authors examined the levels of perfluorooctane sulfonate (PFOS) and perfluorooctanoic acid (PFOA) in different canned fish (35 different products), and all values were within the EU limits. Recent studies indicate that the PFC metabolite trifluoroacetic acid is found in large quantities in surface and groundwater [125], ultimately reaching coastal areas and accumulating in seafood. Therefore, there is an urgent need to assess the accumulation and toxicity levels associated with PFC metabolites in seafood, which can potentially affect the canning industry.

Various studies have reported the contamination of canned seafood products by other POPs. Despite their harmful effects on human health, there are no established regulations for maximum levels. For example, Pye and Crews [126] showed that fish canned with tomato sauce and lemon had the highest furan content, with average values of 0.0049 mg/kg and 0.0055 mg/kg, respectively. In contrast, the furan levels in canned fish in brine or oil were lower than 0.0020 mg/kg, with an average of 0.0002 mg/kg in extra virgin olive oil.

##### Microplastics

Plastics have become ubiquitous in our daily lives due to their resilience, affordability, and unique properties. However, when plastic fragments into smaller particles (e.g., microplastics), it enters the environment, posing a hazard to food systems. In addition to the physical danger, the additives within plastics (approximately 4%) and their ability to adsorb environmental contaminants increase risks to both consumer and ecosystem health [127]. While validated methods exist for quantifying meso- and microplastics in food, no standardized methods exist for nanoplastic assessment [127]. Furthermore, no regulations have been established for limits in food products due to limited information on their occurrence, their toxicity, and the toxicokinetic data required for accurate risk assessment.

Most microplastics in seafood are found in the gastrointestinal tracts, suggesting that gutting fish may reduce human exposure. However, this does not apply to shellfish and small fish species like sardines. Recent studies found higher concentrations of microplastics in the muscle of horse mackerel (63% of specimens), followed by anchovies (40% of specimens), and sardines (39% of specimens), though always being below 100 microplastic particles (mainly blue fibers) per 100 g of muscle [128]. In contrast, the levels of microplastics in mussel meat can reach up to 60 particles per 100 g of tissue [129], while tuna muscle contains lower concentrations (12–27 particles per 100 g) [130]. Seafood is not the only source of plastics; they can originate from various steps along the seafood value chain, including packaging, water, air, machinery, equipment, and textiles. Microplastic levels can also increase during seafood processing. The impact of processes such as nobbing, washing, brining, and heat processing on microplastic content in the canning industry remains poorly studied. Recent findings suggest that fish, additives, and contact materials during cleaning and canning processes contribute significantly to microplastic pollution [131]. Positive correlations have also been observed between salt content and microplastic levels in canned fish, suggesting that salt may be a potential source of microplastics [132].

Research on microplastic levels in canned seafood is limited (see Table 5), and to the authors’ knowledge, no studies have nanoplastic levels in canned seafood. While very few plastic particles are found in the filling medium, microplastics commonly affect most canned products, though with generally low levels (typically 1 to 12 particles per can, but as high as 900 particles per can in extreme cases), regardless of species, filling medium, or type of canning material [13]. A wide array of microplastic polymers, stable under severe heat processing, can be found in canned seafood, except for low-density polyethylene (LDPE), which fully melts and fuses together during the canning steam process [133].

Most studies estimating human intake of microplastics through the consumption of canned seafood suggest that while absorption by consumers is possible, exposure is limited due to the low levels found in products, even among individuals who consume canned seafood several times a week [131,132].

### 3.3. Innovative Canned Fish Products

To simplify everyday meal preparation, canned goods manufacturers are introducing new products that offer nutritional benefits, meeting the growing demand for convenient yet health-conscious food options [134].

The addition of edible macroalgae (e.g., extracts of *Fucus spiralis* or *Bifurcaria bifurcata*) and plant-derived compounds (e.g., cinnamon oil extract) to canned fish products has shown promising results in terms of nutritional, microbial, and sensory quality (Table 6). These findings are detailed in two recent reviews by Aubourg [4] and Gouvêa et al. [135]. Aubourg [4] focused on the impact of adding bioactive compounds to the filling medium on the thermal stability of canned fish. The authors reviewed recent research on the preservative effects and quality impacts of (i) filling medium composition (e.g., water, brine, refined olive oil); (ii) plant-derived compounds added to the filling medium (e.g., baby corn, green pea, broccoli, Indian spice masala mix); (iii) algae-derived compounds as a filling medium (see Table 6); and (iv) seafood by-product compounds (e.g., salmon oil; brine mixed with hydrosol from aromatic plant by-products; octopus cooking liquor). The review primarily focused on inhibiting lipid oxidation and concluded that adding bioactive compounds from natural sources to the filling medium is an effective strategy for producing highly nutritious, safe (e.g., higher n-3 fatty acids, lower thiobarbituric acid reactive substances), and appealing processed products. However, they noted that several factors need to be addressed to enhance the practical and commercial application of this preservation method. In the review by Gouvêa et al. [135], the focus was on using natural antioxidants to manage lipid oxidation in canned fish. The review also examined the antioxidant properties of common filling mediums; the impact of adding algae extracts, herbs, spices, and condiments; and the potential of using food industry by-products (e.g., octopus cooking liquor). The review also highlighted that these practices could positively affect other quality parameters, such as microbiological growth, texture, and water and oil retention capacities.

The recent literature indicates a need for further research to optimize canned fish products, particularly regarding sensory properties for future consumers. This includes studying synergistic combinations of natural materials to enhance quality, such as utilizing underused peptides from food industry co-products. Additionally, research should address the technologies for extracting and preparing these materials, as well as safety considerations, including toxicity studies for new antioxidant sources. Effective concentrations and application methods must also be explored to ensure sensory acceptance by consumers [4,135].

It is important to evaluate the nutritional benefits of new ingredients alongside their potential adverse effects. For example, macroalgae, while a valuable source of nutrients, can also lead to increased exposure to harmful elements like inorganic arsenic, which is carcinogenic, or excessive iodine, which can impair thyroid function due to macroalgae’s high biosorption and accumulation capabilities. Furthermore, since the absorbable quantity of minerals upon ingestion is not accurately predicted by their content in seafood products, it is crucial to quantify their bioaccessibility and bioavailability [140,141,142]. Although iodine from macroalgae is highly bioaccessible, its bioavailability appears to be low [140]. However, there is limited information on iodine bioavailability in macroalgae and macroalgae-fortified foods [141].

Moreover, uncertainties and challenges remain in the commercialization of macroalgae-enriched food products due to their sensory impact and low consumer awareness of their health benefits. Therefore, it is crucial to develop nutritious and healthy products that are also appealing in terms of sensory characteristics. Additionally, regulations on novel foods must be considered [143,144].

## 4. Conclusions and Perspectives

The canned fish sector has achieved positive results in recent years, thanks to technological advances and more responsible attitudes and practices. However, to meet the growing global demand for canned fish, there are challenges that must continue to be considered in order to improve the sector’s performance and respond to consumer and market demands. To meet these requirements, more studies are needed to enable canning companies to fully trace raw materials and processes and thus control and verify the entire supply chain, guaranteeing the quality and safety of canned fish and improving environmental sustainability.

The analysis of contaminants in canned fish products reveals the necessity for stringent regulations and continuous monitoring to uphold consumer safety standards. While many products comply with acceptable limits, instances of elevated levels in specific samples underscore the ongoing need for vigilance in food safety protocols.

Future work should focus on different key areas, namely, (i) developing methods for assessing microplastics and nanoplastics in canned seafood and to better understand their toxicity; (ii) investigating the health effects of BPA substitutes, such as BPS, and exploring safer options; (iii) continuing to monitor and research toxic elements like Cd, Pb, and Hg in canned seafood, especially in raw materials from regions where their concentrations exceed safety limits; and (iv) investigating the efficacy and safety of adding bioactive compounds to canned seafood products, including studies on their bioaccessibility, bioactivity, and consumer acceptance. Strengthening food safety standards, improving transparency and traceability in the production chain, and educating consumers about the benefits and risks of canned products are also crucial. Moreover, addressing these challenges should contribute to improving the sustainability of canned fish production processes.

## Figures and Tables

**Table 1:** Specific Migration Limit (SML, mg/kg food), Maximum Permissible Limits (MPLs, mg/kg food), and Tolerable Daily Intake (TDI, μg/kg bw/day) of BPA and its analogs in canned foods, as established by various countries and organizations.

**Hazard Type**	**Hazard Sub-Type**	**Geographical Range**	**SML/MPLs**	**TDI**	**References**
Biological	Histamine	Europe	200^*^	-	[35,37]
	Histamine	FAO/WHO	200	-	[38]
Chemical	BPA	Europe	0.05	0.002	[10,39,40]
	Σ (BADGE, HD)		9	150	[41,42]
	Σ (BADGE, CD)		1	-	[43]
	BPS		0.05	-	[44]
	CdB		0.05	1.5	[45,46]
	Al	Europe	5	142.9	[47,48,49,50]
	Cd		0.1–0.25	0.36	
	Hg		0.3–1	0.57	
	Pb		0.3	-	
	Sn		200	-	[51]
	Al	FAO/WHO	-	285.7	[52,53]
	Hg		0.5–1	0.57	
	Pb		0.3	-	
	Sn		250	2	
	PAH4	Europe	0.03		[44]
	BaP		0.005		
	Σ dioxins (WHO-PCDD/F-TEQ)		0.0000035		[50]
	Σ dioxins and dioxin-like PCBs (WHO-PCDD/F-PCB-TEQ)		0.0000065–0.00001		
	Σ (PCB28, PCB52, PCB101, PCB138, PCB153, and PCB180 (ICES-6))		0.075–0.300	0.00001	
	PFOS		0.002–0.035		[54]
	PFOA		0.0003–0.008		
	PFNA		0.0007–0.008		
	PFHxS		0.0003–0.0015		
	Σ (PFOS, PFOA, PFNA, and PFHxS)		0.0017–0.045		

* From a set of nine samples, none may exceed 400 mg/kg histamine (“M”), and not more than 2 samples may contain more than 200 mg/kg (“m”). Abbreviations: BPA—Bisphenol A; BADGE—Bisphenol A diglycidyl ether; HD—hydroxyl derivative; CD—Chlorinated derivative; BPS—Bisphenol S; CdB—Cyclo-di-BADGE; Al—Aluminum; Cd—Cadmium; Hg—Mercury; Pb—Lead; Sn—Tin; PAH—Polycyclic aromatic hydrocarbon; BaP—Benzo[a]pyrene; WHO—World Health Organization; PCDD/F—Polychlorinated dibenzo-p-dioxin and furan; TEQ—Toxic equivalent; PCBs—Polychlorinated biphenyls; ICES—International Council for the Exploration of the Sea; PFOS—Perfluorooctane sulfonate; PFOA—Perfluorooctanoic acid; PFNA—Perfluorononanoic acid; PFHxS—perfluorohexanesulphonic acid.

**Table 2:** Concentration range (mg/kg of food) of BPA, BPA analogs, BADGE and its derivatives (HD: hydroxyl derivative; CD: chlorinated derivative), and CdB found in canned fish and seafood from different origins. Limit Of Detection/Quantification (LOD/Q). Filling medium not specified (n.s.). Total number of samples analyzed (*n*).

Origin	Species (Filling Medium)	BPA (*n*)	BPA Analogs		BADGE and Derivatives		CdB (*n*)	References
			BPS (*n*)	Others^a^ (*n*)	Σ[BADGE; HD^b^] (*n*)	Σ[CD^c^] (*n*)		
European countries	Tuna (oil)	<LOD^a^–0.409 (*30*)	<LOD^*^–0.19 (*30*)	<LOD^*^–0.07 (*30*)	<LOD^*^–0.84 (*49*)	<LOD^*^–0.93 (*49*)	<LOQ^*^–0.67 (*28*)	[83,90,91,94,95]
	Tuna (water/brine)	<LOD^*^–0.042 (*11*)	<LOD^*^ (*6*)	<LOD^*^ (*7*)	<LOD^*^–0.51 (*10*)	1.03 (*10*)	0.06–0.34 (*7*)	
Non-European countries	Tuna (n.s.)	0.061–0.200 (*274*_SF^#^)	-	-	-	-	-	[93,96,97]
	Tuna (oil)	0.197–0.198 (*200*_LF^##^)	-	-	-	-	-	
	Tuna (water/brine)	0.197 (*74*_LF^##^)	-	-	-	-	-	
	Fish, squid, and shrimp (n.s.)	0.078 (*4*)	-	0.02 (4)	-	-	-	

^*^ LOD: BPA ≤ 0.001 mg/kg; BPA analogs ≤ 0.002 mg/kg; BADGE and HD ≤ 0.016 mg/kg; CD ≤ 0.017 mg/kg; CdB = 0.001 mg/kg. LOQ: CdB = 0.0125 mg/kg.^a^ BPB (Bisphenol F); BPAF (Bisphenol AF); BPC (Bisphenol F); BPE (Bisphenol E); BPF (Bisphenol E); BPG (Bisphenol G); BPP (Bisphenol P); BPM (Bisphenol M).^b^ BADGE.H2O; BADGE.2H2O.^c^ BADGE HCL; BADGE 2HCL; BADGE H2O HCL.^#^ SF—Solid fraction of product.^##^ LF—Liquid fraction of product.

**Table 3:** Estimated daily intake (μg/kg bw/day) of BPA, BPA analogs, BADGE and its derivatives (HD: hydroxyl derivative; CD: chlorinated derivative), and CdB found in canned fish and seafood from various sources. Filling medium not specified (n.s.).

Origin	Species (Filling Medium)	BPA	BPA Analogs		BADGE and Derivatives		CdB	References
			BPS	Others^a^	Σ[BADGE; HD^b^]	Σ[CD^c^]		
European countries	Tuna (oil)	0.005–0.009	**-**	0.020	0.015	0.020	0.005	[93,94]
	Tuna (water/brine)	0.046	-	0.028	0.553	0.028	0.239	
	Sardines (oil)	0.009	-	0.036	0.027	0.036	0.055	
	Clams (water/brine)	0.005	-	0.020	0.015	0.020	0.005	
	Mussels (pickled)	0.035	-	0.140	0.269	0.140	0.066	
Non-European countries	Tuna (n.s.)	0.006	-	-	-	-	-	[93]

^a^ BPB; BPAF; BPC; BPE; BPF; BPG; BPP; BPM.^b^ BADGE.H2O; BADGE.2H2O.^c^ BADGE HCL; BADGE 2HCL; BADGE H2O HCL.

**Table 5:** Microplastics (MPs) and their levels in canned seafood meat. Polypropylene—PP; polyethylene terephthalate—PET; polyethylene—PE; polyvinyl chloride—PVC; polystyrene—PS; low-density polyethylene—LDPE; polyolefin—POF; polyacrylonitrile—PAN; polymethacrylic acid methyl ester—PMAME; polyamide—PA. Number of samples/brands (*n*).

Species (*n*)	Filling Medium	Frequency of Occurrence (%)	Number of Identified MPs	Type of Plastics	References
Tuna (*14*)	Oil	100	1–12	POF, PAN, PMAME, PA, PET, and PP.	[131]
Tuna (*4*)	Water/Brine	100	3–4		
Skipjack tuna (*5*)	Oil	100	1–6		
Salmon (*3*)	Oil	100	2–6		
Longtail tuna (*20*)	Oil	60–100	2–3	PET, PS, PP, PS-PP, PS-PET, Nylon, PVC, and LDPE.	[132]
Longtail tuna (*5*)	Water/Brine	80	4–5		
Yellowfin tuna (*20*)	Oil	40–100	1–3		
Mackerel (*5*)	Oil	100	3–3		
Sprat (*9* brands)	Oil	22	0–1	PP, PET, PE, and PVC.	[133]
Sardine (*12* brands)	Oil	0	0	-	

**Table 6:** Summary of studies focusing on the development of new formulations and their impact on the quality of canned fish products.

Species	Ingredient Tested	Quantities Tested	Effects	Reference
Mackerel	Aqueous extract of *Fucus spiralis* (ratio: 0.28 of lyophilised alga/5 mL of extract)	5, 15, or 30 mL of extract + 35, 25, or 10 mL of distilled water + 40 mL of brine solution (4% *w*/*v*)	- Free fatty acid content decreased. - Increased peroxide retention. - Reduced fluorescent compounds.	[136]
Mackerel	Dehydrated: *Ascophyllum nodosum* *Fucus spiralis* *Saccorhiza polyschides* *Chondrus crispus* *Porphyra* sp. *Ulva* sp. (ratio: 2 g dw of seaweed/60 g fw of fish)	*C. crispus* and *F. spiralis* were: - added in the canning step (trial A) - boiled with the fish for 20 min and removed after boiling; added new portion in the canning step (trial B)	- Product from trial B was the preferred sensory option.	[137]
Mackerel	Aqueous extracts (brine—aqueous, 2% NaCl medium) of *Fucus spiralis* + *Ulva lactuca* (ratio: 0.56 g of extracted alga/10 mL extract)	10 or 30 mL of each alga extract + 30 or 10 mL of distilled water + 40 mL of brine solution (4% *w*/*v*)	- Loss of lipids after canning inhibited. - Breakdown of fatty acids and peroxides prevented. - Formation of fluorescent compounds reduced.	[138]
Mackerel	Aqueous extract *(water) of Bifurcaria bifurcata* (ratio: 0.625 g of extracted alga/5 mL extract)	5, 10, 25, and 50 mL of alga extract + completed with distilled water	- Inhibitory effect on lipid oxidation development and color parameters.	[23]
Herring Salmon Mackerel	Cinnamon oil extract (which contains a set of fat-soluble substances with a distinct antimicrobial and enzymatic inhibition activity), instead of vegetable/soybean oil	Extract added to the cans: 15% of the net weight	- Cinnamon oil extract: histamine content < 35 mg/kg. - Control (with soybean oil): accumulated histamine ≥ 50 mg/kg.	[139]

Abbreviations: dw—dry weight; fw—fresh weight. NaCl—sodium chloride.

## Data Availability

No new data were created or analyzed in this study. Data sharing is not applicable to this article.

## Abbreviations

Al	Aluminum
BaP	Benzo[a]pyrene
Cd	Cadmium
CdB	Cyclo-di-BADGE
DHA	Docosahexaenoic Acid
DTX	Dinophysistoxin
EPA	Eicosapentaenoic Acid
Hg	Mercury
IgE	Immunoglobulin E
LDPE	Low-Density Polyethylene
LOD	Limit of Detection
LOQ	Limit of Quantification
MPLs	Maximum Permissible Limits
MPs	Microplastics
NaCl	Sodium Chloride
OA	Okadaic Acid
PA	Polyamide
Pb	Lead
PE	Polyethylene
PET	Polyethylene Terephthalate
PFHxS	Perfluorohexane Sulfonate
PFNA	Perfluorononanoic Acid
PFOA	Perfluorooctanoic Acid
PFOS	Perfluorooctane Sulfonate
PMAME	Polymethacrylic Acid Methyl Ester
PP	Polypropylene
PS	Polystyrene
PTX	Pectenotoxin
PVC	Polyvinyl Chloride
PVS	Poly(vinyl stearate)
Sn	Tin
TDI	Tolerable Daily Intake
**Acronyms**	
BADGE	Bisphenol A Diglycidyl Ether
BPA	Bisphenol A
BPAF	Bisphenol AF
BPB	Bisphenol B
BPC	Bisphenol C
BPE	Bisphenol E
BPF	Bisphenol F
BPG	Bisphenol G
BPM	Bisphenol M
BPP	Bisphenol P
BPS	Bisphenol S
EVOH	Ethylene-Vinyl Alcohol
POPs	Persistent Organic Pollutants
**Initialisms**	
CD	Chlorinated Derivative
DST	Diarrhetic Shellfish Toxin
EC	European Commission
EFSA	European Food and Safety Authority
EU	European Union
FAO	Food and Agriculture Organization
HACCP	Hazard Analysis and Critical Control Points
HD	Hydroxyl Derivative
IEAA	Essential Amino Acid Index
LF	Liquid Fraction
n.s.	Not Specified
N/A	Not Applicable
PAHs	Polycyclic Aromatic Hydrocarbons
PAN	Polyacrylonitrile
PCBs	Polychlorinated Biphenyls
PCDD/F-TEQ	Polychlorinated Dibenzo-para-Dioxin/Furan Toxic Equivalence
PFASs	Perfluorinated Alkyl Substances
PFHxS	Perfluorohexane Sulfonate
PFNA	Perfluorononanoic Acid
PFOA	Perfluorooctanoic Acid
PFOS	Perfluorooctane Sulfonate
POF	Polyolefin
PSP	Paralytic Shellfish Poisoning
SF	Solid Fraction
SML	Specific Migration Limit
WHO	World Health Organization

## References

[1] Pitarch J.L., Vilas C., De Prada C., Palacín C.G., Alonso A.A. (2021). Optimal Operation of Thermal Processing of Canned Tuna under Product Variability. J. Food Eng..

[2] Ghaly A.E., Dave D., Budge S., Brooks M.S. (2010). Fish Spoilage Mechanisms and Preservation Techniques: Review. Am. J. Appl. Sci..

[3] Zelasney J., Ford A., Westlund L., Ward A., Riego Peñarubia O. (2020). Securing Sustainable Small-Scale Fisheries: Showcasing Applied Practices in Value Chains, Post-Harvest Operations and Trade.

[4] Aubourg S.P. (2023). Enhancement of Lipid Stability and Acceptability of Canned Seafood by Addition of Natural Antioxidant Compounds to the Packing Medium—A Review. Antioxidants.

[5] Canned Seafood Market Size, Share, Trends & Forecast. 2031. https://www.skyquestt.com/report/canned-seafood-market.

[6] Eissa F., Younes A. (2024). Fish Contamination: Analysis of the EU RASFF Notifications over the Last 23 Years. Food Control.

[7] DeBeeR J., Bell J.W., Nolte F., Arcieri J., Correa G. (2021). Histamine Limits by Country: A Survey and Review. J. Food Prot..

[8] Lambré C., Barat Baviera J.M., Bolognesi C., Chesson A., Cocconcelli P.S., Crebelli R., Gott D.M., Grob K., Lampi E., EFSA Panel on Food Contact Materials, Enzymes and Processing Aids (CEP) (2023). Re-evaluation of the Risks to Public Health Related to the Presence of Bisphenol A (BPA) in Foodstuffs. EFS2.

[9] European Commission (2023). Commission Regulation (EU) (Draft) on the Use of Bisphenol A (BPA) and Other Bisphenols and Their Derivatives with Harmonised Classification for Specific Hazardous Properties in Certain Materials and Articles Intended to Come into Contact with Food, Amending Regulation (EU) No 10/2011, Amending Regulation (EC) No 1895/2005 and Repealing Regulation (EU) 2018/213.

[10] European Commission (2023). Questions and Answers (Q&A) Concerning the Risk Management Approach for Bisphenol A (BPA) and Other Bisphenols in Food Contact Materials (FCMs).

[11] Mahmudiono T., Fakhri Y., Adiban M., Sarafraz M., Mohamadi S. (2024). Concentration of Potential Toxic Elements in Canned Tuna Fish: Systematic Review and Health Risk Assessment. Int. J. Environ. Health Res..

[12] Rúbies A., Muñoz E., Gibert D., Cortés-Francisco N., Granados M., Caixach J., Centrich F. (2015). New Method for the Analysis of Lipophilic Marine Biotoxins in Fresh and Canned Bivalves by Liquid Chromatography Coupled to High Resolution Mass Spectrometry: A Quick, Easy, Cheap, Efficient, Rugged, Safe Approach. J. Chromatogr. A.

[13] Diaz-Basantes M.F., Nacimba-Aguirre D., Conesa J.A., Fullana A. (2022). Presence of Microplastics in Commercial Canned Tuna. Food Chem..

[14] Aksun Tümerkan E.T. (2022). Detection of Parvalbumin Fish Allergen in Canned Tuna by Real-Time PCR Driven by Tuna Species and Can-Filling Medium. Molecules.

[15] DG MARE (2019). Evaluation of the Marketing Standards Framework for Fishery and Aquaculture Products. Specific Contract No. 5 under Framework Contract EASME/EMFF/2016/029. Final Report.

[16] Bavinck M., Ahern M., Hapke H.M., Johnson D.S., Kjellevold M., Kolding J., Overå R., Schut T., Franz N. (2023). Small Fish for Food Security and Nutrition.

[17] Ali A., Wei S., Ali A., Khan I., Sun Q., Xia Q., Wang Z., Han Z., Liu Y., Liu S. (2022). Research Progress on Nutritional Value, Preservation and Processing of Fish—A Review. Foods.

[18] Tavares J., Martins A., Fidalgo L.G., Lima V., Amaral R.A., Pinto C.A., Silva A.M., Saraiva J.A. (2021). Fresh Fish Degradation and Advances in Preservation Using Physical Emerging Technologies. Foods.

[19] Prego R., Trigo M., Martínez B., Aubourg S.P. (2022). Effect of Previous Frozen Storage, Canning Process and Packing Medium on the Fatty Acid Composition of Canned Mackerel. Mar. Drugs.

[20] Campos C.A., Gliemmo M.F., Aubourg S.P., Velázquez J.B., McElhatton A., do Amaral Sobral P.J. (2012). Novel Technologies for the Preservation of Chilled Aquatic Food Products. Novel Technologies in Food Science: Their Impact on Products, Consumer Trends and the Environment.

[21] Fardella M., Ramírez C., Caballero E., Sánchez E., Pinto M., Núñez H., Valencia P., Almonacid S., Simpson R. (2021). Variable Retort Temperature Profiles (VRTPs) and Retortable Pouches as Tools to Minimize Furan Formation in Thermally Processed Food. Foods.

[22] Simpson R., Jiménez D., Almonacid S., Nuñez H., Pinto M., Ramírez C., Vega-Castro O., Fuentes L., Angulo A. (2020). Assessment and Outlook of Variable Retort Temperature Profiles for the Thermal Processing of Packaged Foods: Plant Productivity, Product Quality, and Energy Consumption. J. Food Eng..

[23] Barbosa R.G., Trigo M., Fett R., Aubourg S.P. (2018). Impact of a Packing Medium with Alga Bifurcaria Bifurcata Extract on Canned Atlantic Mackerel (Scomber Scombrus) Quality. J. Sci. Food Agric..

[24] Lahamy A.A.E., Mohamed H.R. (2020). Changes in Fish Quality During Canning Process and Storage Period of Canned Fish Products: Review Article. Glob. J. Nutr. Food Sci..

[25] Malga J.M., Trigo M., Martínez B., Aubourg S.P. (2022). Preservative Effect on Canned Mackerel (*Scomber colias*) Lipids by Addition of Octopus (*Octopus vulgaris*) Cooking Liquor in the Packaging Medium. Molecules.

[26] Reblová Z., Aubourg S.P., Pokorný J. (2022). The Effect of Different Freshness of Raw Material on Lipid Quality and Sensory Acceptance of Canned Sardines. Foods.

[27] Prego R., Fidalgo L.G., Saraiva J.A., Vázquez M., Aubourg S.P. (2021). Impact of Prior High-Pressure Processing on Lipid Damage and Volatile Amines Formation in Mackerel Muscle Subjected to Frozen Storage and Canning. LWT.

[28] Domiszewski Z. (2021). Effect of Sterilization on True Retention Rate of Eicosapentaenoic and Docosahexaenoic Acid Content in Mackerel (*Scomber scombrus*), Herring (*Clupea Harengus*), and Sprat (*Sprattus sprattus*) Canned Products. J. Food Process. Preserv..

[29] Mohan C.O., Remya S., Murthy L.N., Ravishankar C.N., Asok Kumar K. (2015). Effect of Filling Medium on Cooking Time and Quality of Canned Yellowfin Tuna (*Thunnus albacares*). Food Control.

[30] Vinagre J., Rodríguez A., Larraín M.A., Aubourg S.P. (2011). Chemical Composition and Quality Loss during Technological Treatment in Coho Salmon (*Oncorhynchus kisutch*). Food Res. Int..

[31] Naseri M., Rezaei M. (2012). Lipid Changes During Long-Term Storage of Canned Sprat. J. Aquat. Food Prod. Technol..

[32] Gómez-Limia L., Cobas N., Franco I., Martínez-Suárez S. (2020). Fatty Acid Profiles and Lipid Quality Indices in Canned European Eels: Effects of Processing Steps, Filling Medium and Storage. Food Res. Int..

[33] Dantas N.M., De Oliveira V.S., Sampaio G.R., Chrysostomo Y.S.K., Chávez D.W.H., Gamallo O.D., Sawaya A.C.H.F., Torres E.A.F.D.S., Saldanha T. (2021). Lipid Profile and High Contents of Cholesterol Oxidation Products (COPs) in Different Commercial Brands of Canned Tuna. Food Chem..

[34] Samarajeewa U. (2023). Emerging Challenges in Maintaining Marine Food-Fish Availability and Food Safety. Compr. Rev. Food Sci. Food Saf..

[35] European Commission (2005). Commission Regulation (EC) No 2073/2005 of 15 November 2005 on Microbiological Criteria for Foodstuffs.

[36] Mercogliano R., Santonicola S. (2019). Scombroid Fish Poisoning: Factors Influencing the Production of Histamine in Tuna Supply Chain. A Review. LWT.

[37] European Union (2013). Commission Regulation (EU) No 1019/2013 of 23 October 2013 Amending Annex I to Regulation (EC) No 2073/2005 as Regards Histamine in Fishery productstext with EEA Relevance.

[38] FAO (2018). WHO Revision of the Code of Practice for Fish and Fishery Products (Cxc_52_3002) and Revisions of the Section on Sampling, Examination and Analyses Related to Histamine Food Safety.

[39] European Union (2018). Commission Regulation (EU) 2018/213 of 12 February 2018 on the Use of Bisphenol A in Varnishes and Coatings Intended to Come into Contact with Food and Amending Regulation (EU) No 10 /2011 as Regards the Use of That Substance in Plastic Food Contact Materials.

[40] EFSA CEF Panel (EFSA Panel on Food Contact Materials, Enzymes, Flavourings and Processing Aids) (2015). Scientific Opinion on the Risks to Public Health Related to the Presence of Bisphenol A (BPA) in Foodstuffs: Executive Summary. EFSA J..

[41] European Commission (2002). Commission Directive 2002/16/EC of 20 February 2002 on the Use of Certain. Epoxy Derivatives in Materials and Articles Intended to Come into Contact with Foodstuffs.

[42] European Food Safety Authority (EFSA) (2004). Opinion of the Scientific Panel on Food Additives, Flavourings, Processing Aids and Materials in Contact with Food (AFC) Related to 2,2-Bis(4-Hydroxyphenyl)Propane Bis(2,3-Epoxypropyl)Ether (Bisphenol A Diglycidyl Ether, BADGE). REF. No 13510 and 39700. EFSA J..

[43] European Commission (2005). Commission Regulation (EC) No 1895/2005 of 18 November 2005 on the Restriction of Use of Certain Epoxy Derivatives in Materials and Articles Intended to Come into Contact with Food.

[44] European Union (2011). Commission Regulation (EU) No 835/2011 of 19 August 2011 Amending Regulation (EC) No 1881/2006 as Regards Maximum Levels for Polycyclic Aromatic Hydrocarbons in Foodstuffstext with EEA Relevance.

[45] BfR (2016). Epoxide Resin Coatings of Cans: Substance Transfer to Oil-Containing Foods Possible. https://www.bfr.bund.de/cm/349/epoxide-resin-coatings-of-cans-substance-transfer-to-oil-containing-foods-possible.pdf.

[46] Biedermann S., Zurfluh M., Grob K., Vedani A., Brüschweiler B.J. (2013). Migration of Cyclo-diBA from Coatings into Canned Food: Method of Analysis, Concentration Determined in a Survey and in Silico Hazard Profiling. Food Chem. Toxicol..

[47] (2008). EFSA Scientific Opinion of the Panel on Food Additives, Flavourings, Processing Aids and Food Contact Materials on a Request from European Commission on Safety of Aluminium from Dietary Intake. EFSA J..

[48] EFSA Panel on Contaminants in the Food Chain (CONTAM) (2011). Statement on Tolerable Weekly Intake for Cadmium. EFS2.

[49] EFSA Panel on Contaminants in the Food Chain (CONTAM) (2012). Scientific Opinion on the Risk for Public Health Related to the Presence of Mercury and Methylmercury in Food. EFS2.

[50] European Commission (2023). Commission Regulation (EU) 2023/915 of 25 April 2023 on Maximum Levels for Certain Contaminants in Food and Repealing Regulation (EC) No 1881/2006.

[51] European Commission (2006). Commission Regulation (EC) No 1881/2006 of 19 December 2006 Setting Maximum Levels for Certain Contaminants in Foodstuffs.

[52] FAO (2023). WHO General Standard for Contaminants and Toxins in Food and Feed. CXS 193-1995.

[53] Joint FAO/WHO Expert Committee on Food Additives (2011). Evaluation of Certain Food Additives and Contaminants: Seventy-Fourth [74th] Report of the Joint FAO/WHO Expert Committee on Food Additives.

[54] European Union (2022). Commission Regulation (EU) 2022/2388 of 7 December 2022 Amending Regulation (EC) No 1881/2006 as Regards Maximum Levels of Perfluoroalkyl Substances in Certain Foodstuffs.

[55] Elbayoumi Z.H., Dawod E.E., Shawish R.R. (2023). Occurrence and Control of Biogenic Amines in Fresh Fish and Products of Fish. J. Adv. Vet. Res..

[56] Nagy N., Kirrella G.A.K., Moustafa N.Y., Abdallah R. (2023). Quality Assessment of Some Imported and Local Canned Tuna Sold in Kafrelsheikh, Egypt. J. Adv. Vet. Res..

[57] Harmoko H., Kartasasmita R.E., Munawar H., Rakhmawati A., Budiawan B. (2022). Determination of Histamine in Different Compositions of Commercially Canned Fish in Indonesia by Modified QuEChERS and LC-MS/MS. J. Food Compos. Anal..

[58] Crobu L., Mudadu A.G., Melillo R., Pais G.L., Meloni D. (2021). Qualitative Determination of Histamine in Canned Yellowfin Tuna (*Thunnus Albacares*) Marketed in Sardinia (Italy) by Rapid Screening Methods. Ital J Food Saf..

[59] Lo Magro S., Summa S., Iammarino M., D’Antini P., Marchesani G., Chiaravalle A., Muscarella M. (2020). A 5-Years (2015–2019) Control Activity of an EU Laboratory: Contamination of Histamine in Fish Products and Exposure Assessment. Appl. Sci..

[60] Peivasteh-Roudsari L., Rahmani A., Shariatifar N., Tajdar-Oranj B., Mazaheri M., Sadighara P., Khaneghah A.M. (2020). Occurrence of Histamine in Canned Fish Samples (Tuna, Sardine, Kilka, and Mackerel) from Markets in Tehran. J. Food Prot..

[61] Weremfo A., Eduafo M.K., Gyimah H.A., Abassah-Oppong S. (2020). Monitoring the Levels of Biogenic Amines in Canned Fish Products Marketed in Ghana. J. Food Qual..

[62] Pavlović M., Ivanović S., Pavlović I., Rokvić N., Radosavljević V., Vasilev D. (2019). Histamine Levels in Fish Samples Collected from Serbian Market in 2018. Food Feed Res..

[63] El Hariri O., Bouchriti N., Bengueddour R. (2018). Risk Assessment of Histamine in Chilled, Frozen, Canned and Semi-Preserved Fish in Morocco; Implementation of Risk Ranger and Recommendations to Risk Managers. Foods.

[64] Rahmani J., Miri A., Mohseni-Bandpei A., Fakhri Y., Bjørklund G., Keramati H., Moradi B., Amanidaz N., Shariatifar N., Khaneghah A.M. (2018). Contamination and Prevalence of Histamine in Canned Tuna from Iran: A Systematic Review, Meta-Analysis, and Health Risk Assessment. J. Food Prot..

[65] Yesudhason P., Al-Zidjali M., Al-Zidjali A., Al-Busaidi M., Al-Waili A., Al-Mazrooei N., Al-Habsi S. (2013). Histamine Levels in Commercially Important Fresh and Processed Fish of Oman with Reference to International Standards. Food Chem..

[66] Silva T.M., Sabaini P.S., Evangelista W.P., Gloria M.B.A. (2011). Occurrence of Histamine in Brazilian Fresh and Canned Tuna. Food Control.

[67] Li C., Vrdoljak G., Moezzi B. (2018). Sampling and Analysis of Histamine in Fish Products from Local Northern California Markets. Food Nutr. J..

[68] Sadeghi N., Behzad M., Jannat B., Oveisi M.R., Hajimahmoodi M., Mozafazri M. (2020). Determination of Histamine in Canned Tuna Fish Available in Tehran Market by ELISA Method. J. Food Saf. Hyg..

[69] Er B., Demirhan B., Bas S.Y., Yentur G., Oktem A.B. (2014). Determination of Histamine Levels in Canned Tuna Fish. Bulg. J. Agric. Sci..

[70] Kuehn A., Swoboda I., Arumugam K., Hilger C., Hentges F. (2014). Fish Allergens at a Glance: Variable Allergenicity of Parvalbumins, the Major Fish Allergens. Front. Immunol..

[71] Liang J., Taylor S.L., Baumert J., Lopata A.L., Lee N.A. (2021). Effects of Thermal Treatment on the Immunoreactivity and Quantification of Parvalbumin from Southern Hemisphere Fish Species with Two Anti-Parvalbumin Antibodies. Food Control.

[72] Blickem E.R., Bell J.W., Baumgartel D.M., Debeer J. (2022). Review and Analysis of Tuna Recalls in the United States, 2002 through 2020. J. Food Prot..

[73] Taki A.C., Ruethers T., Nugraha R., Karnaneedi S., Williamson N.A., Nie S., Leeming M.G., Mehr S.S., Campbell D.E., Lopata A.L. (2023). Thermostable Allergens in Canned Fish: Evaluating Risks for Fish Allergy. Allergy.

[74] Pecoraro L., Infante S., Fuentes-Aparicio V., Cabrera-Freitag P., Antonucci N., Alvarez-Perea A. (2021). IgE-mediated Fish Allergy in Pediatric Age: Does Canned Tuna Have a Chance for Tolerance?. Pediatr. Allergy Immunol..

[75] Nielsen L.T., Hansen P.J., Krock B., Vismann B. (2016). Accumulation, Transformation and Breakdown of DSP Toxins from the Toxic Dinoflagellate Dinophysis Acuta in Blue Mussels, Mytilus Edulis. Toxicon.

[76] Leite I.D.P., Sdiri K., Taylor A., Viallon J., Gharbia H.B., Mafra Júnior L.L., Swarzenski P., Oberhaensli F., Darius H.T., Chinain M. (2021). Experimental Evidence of Ciguatoxin Accumulation and Depuration in Carnivorous Lionfish. Toxins.

[77] European Economic Community E.E.C. (1991). Council Directive of 15 July 1991 Laying down the Health Conditions for the Production and the Placing on the Market of Live Bivalve Molluscs. Off. J. Eur. Union.

[78] European Commission (2004). Directive 2004/41/EC of the European Parliament and of the Council of 21 April 2004 Repealing Certain Directives Concerning Food Hygiene and Health Conditions for the Production and Placing on the Market of Certain Products of Animal Origin Intended for Human Consumption and Amending Council Directives 89/662/EEC and 92/118/EEC and Council Decision 95/408/EC.

[79] Blanco J., Arévalo F., Correa J., Porro M.C., Cabado A.G., Vieites J.M., Moroño A. (2016). Effect of the Industrial Canning on the Toxicity of Mussels Contaminated with Diarrhetic Shellfish Poisoning (DSP) Toxins. Toxicon.

[80] Rodríguez I., Alfonso A., Antelo A., Alvarez M., Botana L. (2016). Evaluation of the Impact of Mild Steaming and Heat Treatment on the Concentration of Okadaic Acid, Dinophysistoxin-2 and Dinophysistoxin-3 in Mussels. Toxins.

[81] García C., Oyaneder-Terrazas J., Contreras C., Del Campo M., Torres R., Contreras H.R. (2016). Determination of the Toxic Variability of Lipophilic Biotoxins in Marine Bivalve and Gastropod Tissues Treated with an Industrial Canning Process. Food Addit. Contam. Part A.

[82] LaKind J.S. (2013). Can Coatings for Foods and Beverages: Issues and Options. Int. J. Technol. Policy Manag..

[83] Toptancı İ., Kıralan M., Ketenoglu O., Ramadan M.F. (2022). Monitoring of Bisphenol A Diglycidyl Ether (BADGE) and Some Derivatives in Fish Products in the Turkey Market. Environ. Sci. Pollut. Res..

[84] Cunha S.C., Fernandes J.O. (2013). Assessment of Bisphenol A and Bisphenol B in Canned Vegetables and Fruits by Gas Chromatography–Mass Spectrometry after QuEChERS and Dispersive Liquid–Liquid Microextraction. Food Control.

[85] Beausoleil C., Emond C., Cravedi J.-P., Antignac J.-P., Applanat M., Appenzeller B.R., Beaudouin R., Belzunces L.P., Canivenc-Lavier M.-C., Chevalier N. (2018). Regulatory Identification of BPA as an Endocrine Disruptor: Context and Methodology. Mol. Cell. Endocrinol..

[86] Ohore O.E., Zhang S. (2019). Endocrine Disrupting Effects of Bisphenol A Exposure and Recent Advances on Its Removal by Water Treatment Systems. A Review. Sci. Afr..

[87] Wang D., Zhao H., Fei X., Synder S.A., Fang M., Liu M. (2021). A Comprehensive Review on the Analytical Method, Occurrence, Transformation and Toxicity of a Reactive Pollutant: BADGE. Environ. Int..

[88] Catenza C.J., Farooq A., Shubear N.S., Donkor K.K. (2021). A Targeted Review on Fate, Occurrence, Risk and Health Implications of Bisphenol Analogues. Chemosphere.

[89] Geens T., Aerts D., Berthot C., Bourguignon J.-P., Goeyens L., Lecomte P., Maghuin-Rogister G., Pironnet A.-M., Pussemier L., Scippo M.-L. (2012). A Review of Dietary and Non-Dietary Exposure to Bisphenol-A. Food Chem. Toxicol..

[90] Toptancı İ. (2023). Risk Assessment of Bisphenol Related Compounds in Canned Convenience Foods, Olives, Olive Oil, and Canned Soft Drinks in Turkey. Environ. Sci. Pollut. Res..

[91] Gálvez-Ontiveros Y., Moscoso-Ruiz I., Rodrigo L., Aguilera M., Rivas A., Zafra-Gómez A. (2021). Presence of Parabens and Bisphenols in Food Commonly Consumed in Spain. Foods.

[92] Repossi A., Farabegoli F., Gazzotti T., Zironi E., Pagliuca G. (2016). Bisphenol A in Edible Part of Seafood. Ital. J. Food Saf..

[93] Al Ghoul L., Abiad M.G., Jammoul A., Matta J., El Darra N. (2020). Zinc, Aluminium, Tin and Bis-Phenol a in Canned Tuna Fish Commercialized in Lebanon and Its Human Health Risk Assessment. Heliyon.

[94] Lestido-Cardama A., Sendón R., Bustos J., Santillana M.I., Paseiro Losada P., Rodríguez Bernaldo De Quirós A. (2021). Multi-Analyte Method for the Quantification of Bisphenol Related Compounds in Canned Food Samples and Exposure Assessment of the Spanish Adult Population. Food Packag. Shelf Life.

[95] Maragou N.C., Thomaidis N.S., Theodoridis G.A., Lampi E.N., Koupparis M.A. (2020). Determination of Bisphenol A in Canned Food by Microwave Assisted Extraction, Molecularly Imprinted Polymer-Solid Phase Extraction and Liquid Chromatography-Mass Spectrometry. J. Chromatogr. B.

[96] Shaaban H., Mostafa A., Alqarni A.M., Almohamed Y., Abualrahi D., Hussein D., Alghamdi M. (2022). Simultaneous Determination of Bisphenol A and Its Analogues in Foodstuff Using UPLC-MS/MS and Assessment of Their Health Risk in Adult Population. J. Food Compos. Anal..

[97] Osman M.A., Mahmoud G.I., Elgammal M.H., Hasan R.S. (2018). Studying of Bisphenol A Levels in Some Canned Food, Feed and Baby Bottles in Egyptian Markets. Fresenius Environ. Bull..

[98] Morshdy A.E., Hussein M., Darwish W., Yousef R., Tharwat A. (2021). Residual Contents of Selected Heavy Metals in Commercial Canned Fish in Egypt: Dietary Intakes and Health Risk Assessment. Slov. Vet. Res..

[99] Inan-Eroglu E., Ayaz A. (2018). Is Aluminum Exposure a Risk Factor for Neurological Disorders?. J. Res. Med Sci..

[100] Mol S. (2011). Levels of Heavy Metals in Canned Bonito, Sardines, and Mackerel Produced in Turkey. Biol. Trace Element Res..

[101] Kosker A.R., Gundogdu S., Esatbeyoglu T., Ayas D., Ozogul F. (2023). Metal Levels of Canned Fish Sold in Türkiye: Health Risk Assessment. Front. Nutr..

[102] de Lima N.V., Melo E.S.D.P., Arakaki D.G., Tschinkel P.F.S., De Souza I.D., Ulbrecht M.O.D.O., Mendes Dos Reis F.J., Rosa A.C.G., Rosa R.H., Aragão Do Nascimento V. (2021). Data on Metals, Nonmetal, and Metalloid in the Samples of the Canned Tuna and Canned Sardines Sold in Brazil. Data Brief.

[103] Ababneh F.A., Al-Momani I.F. (2013). Levels of Mercury, Cadmium, Lead and Other Selected Elements in Canned Tuna Fish Commercialised in Jordan. Int. J. Environ. Anal. Chem..

[104] Massadeh A.M., Al-Massaedh A.A.T., Kharibeh S. (2018). Determination of Selected Elements in Canned Food Sold in Jordan Markets. Environ. Sci. Pollut. Res..

[105] Sadighara P., Mofid V., Mahmudiono T., Rahmani A., Tajdar-Oranj B., Peivasteh-Roudsari L., Farhangfar A., Moradi M., Fakhri Y. (2022). Concentration of Heavy Metals in Canned Tuna Fish and Probabilistic Health Risk Assessment in Iran. Int. J. Environ. Anal. Chem..

[106] Sobhanardakani S. (2017). Tuna Fish and Common Kilka: Health Risk Assessment of Metal Pollution through Consumption of Canned Fish in Iran. J. Consum. Prot. Food Saf..

[107] Mansouri B., Azadi N.A., Albrycht M., Binkowski L.J., Błaszczyk M., Hamesadeghi U., Rahmani R., Maleki A., Majnoni F. (2021). Metal Risk Assessment Study of Canned Fish Available on the Iranian Market. Biol. Trace Element Res..

[108] Núñez R., García M.Á., Alonso J., Melgar M.J. (2018). Arsenic, Cadmium and Lead in Fresh and Processed Tuna Marketed in Galicia (NW Spain): Risk Assessment of Dietary Exposure. Sci. Total Environ..

[109] Popovic A.R., Djinovic-Stojanovic J.M., Djordjevic D.S., Relic D.J., Vranic D.V., Milijasevic M.P., Pezo L.L. (2018). Levels of Toxic Elements in Canned Fish from the Serbian Markets and Their Health Risks Assessment. J. Food Compos. Anal..

[110] Novakov N.J., Mihaljev Ž.A., Kartalović B.D., Blagojević B.J., Petrović J.M., Ćirković M.A., Rogan D.R. (2017). Heavy Metals and PAHs in Canned Fish Supplies on the Serbian Market. Food Addit. Contam. Part B.

[111] Andayesh S., Hadiani M.R., Mousavi Z., Shoeibi S. (2015). Lead, Cadmium, Arsenic and Mercury in Canned Tuna Fish Marketed in Tehran, Iran. Food Addit. Contam. Part B.

[112] de Mello Lazarini T.E., Milani R.F., Yamashita D.M., Saron E.S., Morgano M.A. (2019). Canned Sardines Commercialized in Brazil: Packaging and Inorganic Contaminants Evaluation. Food Packag. Shelf Life.

[113] Rahimi E., Hajisalehi M., Kazemeini H.R., Chakeri A., Derakhshesh M., Mirdamadi M., Ebadi A.G., Rezvani A., Kashkahi M.F. (2010). Analysis and Determination of Mercury, Cadmium and Lead in Canned Tuna Fish Marketed in Iran. Afr. J. Biotechnol..

[114] Huang W., Huang Y., Chen Y., Tan W., Wu K. (2023). A Comprehensive Review of the Human Body Burden of Persistent Organic Pollutants (POPs) and Associated Health Effects in an e-Waste Recycling Area in China. Discov. Environ..

[115] Singh L., Agarwal T. (2018). Polycyclic Aromatic Hydrocarbons in Diet: Concern for Public Health. Trends Food Sci. Technol..

[116] Benson N.U., Anake W.U., Adedapo A.E., Fred-Ahmadu O.H., Eke K.P. (2017). Polycyclic Aromatic Hydrocarbons in Imported Sardinops Sagax: Levels and Health Risk Assessments through Dietary Exposure in Nigeria. J. Food Compos. Anal..

[117] Zachara A., Gałkowska D., Juszczak L. (2017). Contamination of Smoked Meat and Fish Products from Polish Market with Polycyclic Aromatic Hydrocarbons. Food Control.

[118] Drabova L., Pulkrabova J., Kalachova K., Tomaniova M., Kocourek V., Hajslova J. (2013). Polycyclic Aromatic Hydrocarbons and Halogenated Persistent Organic Pollutants in Canned Fish and Seafood Products: Smoked versus Non-Smoked Products. Food Addit. Contam. Part A.

[119] El Morsy F.A.M., El-Sadaawy M.M., Ahdy H.H.H., Abdel-Fattah L.M., El-Sikaily A.M., Khaled A., Tayel F.M.T. (2013). Potential Human Health Risks from Toxic Metals, Polycyclic Aromatic Hydrocarbons, Polychlorinated Biphenyls, and Organochlorine Pesticides via Canned Fish Consumption: Estimation of Target Hazard Quotients. J. Environ. Sci. Health Part A.

[120] Iwegbue C.M.A., Overah L.C., Tesi G.O., Bassey F.I., Martincigh B.S. (2015). Polycyclic Aromatic Hydrocarbon Profiles of Some Brands of Canned Fish in the Nigerian Market. Hum. Ecol. Risk Assess. Int. J..

[121] Johnson L.L., Anulacion B.F., Arkoosh M.R., Burrows D.G., Da Silva D.A.M., Dietrich J.P., Myers M.S., Spromberg J., Ylitalo G.M. (2013). Effects of Legacy Persistent Organic Pollutants (POPs) in Fish—Current and Future Challenges. Fish Physiology.

[122] Afolabi O.L., Iwegbue C.M.A., Obi G., Tesi G.O., Nwajei G.E., Martincigh B.S. (2023). Polychlorinated Biphenyls and Polychlorinated Dibenzo-p-Dioxins and Furans in Imported Canned Fish in Nigeria and Risk Assessment. Food Addit. Contam. Part B.

[123] Vali Mohammadi F., Qajarbeygi P., Shariatifar N., Mahmoudi R., Arabameri M. (2023). Measurement of Polychlorinated Biphenyls in Different High Consumption Canned Foods, Using the QuEChERS/GC-MS Method. Food Chem. X.

[124] Hrádková P., Poustka J., Hloušková V., Pulkrabová J., Tomaniová M., Hajšlová J. (2010). Perfluorinated Compounds: Occurrence of Emerging Food Contaminants in Canned Fish and Seafood Products. Czech J. Food Sci..

[125] TFA in Water: Dirty PFAS Legacy Under the Radar. https://www.pan-europe.info/resources/reports/2024/05/tfa-water-dirty-pfas-legacy-under-radar.

[126] Pye C., Crews C. (2014). Furan in Canned Sardines and Other Fish. Food Addit. Contam. Part B.

[127] (2016). EFSA Panel on Contaminants in the Food Chain (CONTAM) Presence of Microplastics and Nanoplastics in Food, with Particular Focus on Seafood. EFSA J..

[128] Lopes C., Ambrosino A.C., Figueiredo C., Caetano M., Santos M.M., Garrido S., Raimundo J. (2023). Microplastic Distribution in Different Tissues of Small Pelagic Fish of the Northeast Atlantic Ocean. Sci. Total Environ..

[129] Nalbone L., Cincotta F., Giarratana F., Ziino G., Panebianco A. (2021). Microplastics in Fresh and Processed Mussels Sampled from Fish Shops and Large Retail Chains in Italy. Food Control.

[130] Di Giacinto F., Di Renzo L., Mascilongo G., Notarstefano V., Gioacchini G., Giorgini E., Bogdanović T., Petričević S., Listeš E., Brkljača M. (2023). Detection of Microplastics, Polymers and Additives in Edible Muscle of Swordfish (*Xiphias gladius*) and Bluefin Tuna (*Thunnus thynnus*) Caught in the Mediterranean Sea. J. Sea Res..

[131] Gündoğdu S., Köşker A.R. (2023). Microplastic Contamination in Canned Fish Sold in Türkiye. PeerJ.

[132] Akhbarizadeh R., Dobaradaran S., Nabipour I., Tajbakhsh S., Darabi A.H., Spitz J. (2020). Abundance, Composition, and Potential Intake of Microplastics in Canned Fish. Mar. Pollut. Bull..

[133] Karami A., Golieskardi A., Choo C.K., Larat V., Karbalaei S., Salamatinia B. (2018). Microplastic and Mesoplastic Contamination in Canned Sardines and Sprats. Sci. Total Environ..

[134] Carlucci D., Nocella G., De Devitiis B., Viscecchia R., Bimbo F., Nardone G. (2015). Consumer Purchasing Behaviour towards Fish and Seafood Products. Patterns and Insights from a Sample of International Studies. Appetite.

[135] Gouvêa F.D.J., De Oliveira V.S., Mariano B.J., Takenaka N.A.R., Gamallo O.D., Da Silva Ferreira M., Saldanha T. (2023). Natural Antioxidants as Strategy to Minimize the Presence of Lipid Oxidation Products in Canned Fish: Research Progress, Current Trends and Future Perspectives. Food Res. Int..

[136] Aubourg S.P., Trigo M., Martínez B., Rodríguez A. (2020). Effect of Prior Chilling Period and Alga-Extract Packaging on the Quality of a Canned Underutilised Fish Species. Foods.

[137] Vieira E.F., Soares C., Machado S., Oliva-Teles M.T., Correia M., João Ramalhosa M., Carvalho A., Domingues V.F., Antunes F., Morais S. (2020). Development of New Canned Chub Mackerel Products Incorporating Edible Seaweeds—Influence on the Minerals and Trace Elements Composition. Molecules.

[138] Barbosa R.G., Trigo M., Campos C.A., Aubourg S.P. (2019). Preservative Effect of Algae Extracts on Lipid Composition and Rancidity Development in Brine-Canned Atlantic Chub Mackerel (*Scomber colias*). Eur. J. Lipid Sci. Technol..

[139] Shulgin Y.P., Lazhentseva L.Y., Shulgina L.V., Kalenik T.K., Matveeva V.A., Piekoszewski W. (2017). Quality Improvement of Canned Fish with the Use of Cinnamon Oil Extract. Int. J. Food Eng..

[140] Circuncisão A.R., Catarino M.D., Cardoso S.M., Silva A.M.S. (2018). Minerals from Macroalgae Origin: Health Benefits and Risks for Consumers. Mar. Drugs.

[141] Afonso N.C., Catarino M.D., Silva A.M.S., Cardoso S.M. (2019). Brown Macroalgae as Valuable Food Ingredients. Antioxidants.

[142] Sá Monteiro M., Sloth J., Holdt S., Hansen M., National Food Institute, Technical University of Denmark, Denmark (2019). Analysis and Risk Assessment of Seaweed. EFS2.

[143] Roohinejad S., Koubaa M., Barba F.J., Saljoughian S., Amid M., Greiner R. (2017). Application of Seaweeds to Develop New Food Products with Enhanced Shelf-Life, Quality and Health-Related Beneficial Properties. Food Res. Int..

[144] Lähteenmäki-Uutela A., Rahikainen M., Camarena-Gómez M.T., Piiparinen J., Spilling K., Yang B. (2021). European Union Legislation on Macroalgae Products. Aquac. Int..

